# Chemical Composition and Biological Activity of Phenolic Compounds and Carotenoids Extracted from Yellow-Purple Polignano Carrots

**DOI:** 10.3390/foods15091586

**Published:** 2026-05-04

**Authors:** Maria Cefola, Stefania Moccia, Virginia Carbone, Paola Minasi, Bernardo Pace, Michela Palumbo, Gian Luigi Russo, Maria Russo

**Affiliations:** 1 Institute of Sciences of Food Production, National Research Council (CNR), Via Michele Protano c/o CS-DAT, 71121 Foggia, Italy; maria.cefola@cnr.it (M.C.); bernardo.pace@cnr.it (B.P.);; 2 Institute of Food Sciences, National Research Council (CNR), Via Roma 64, 83100 Avellino, Italy; stefania.moccia@isa.cnr.it (S.M.); gianluigi.russo@cnr.it (G.L.R.)

**Keywords:** *Daucus carota* L., (poly)phenols, carotenoid nanoemulsion, antioxidant activity, apoptosis, human cell lines

## Abstract

Anthocyanin-rich fruits and vegetables are widely recognized for their antioxidant and health-promoting properties. This study investigated the chemical composition and in vitro biological activity of extracts from the yellow-purple Polignano carrot, a traditional multicolored landrace from Southern Italy. Among the yellow, orange, and purple variants, the purple carrots exhibited an approximately tenfold higher antioxidant capacity (45 ± 0.23 mg Trolox g^−1^ FW) and were therefore selected for further analysis. Fifteen phenolic compounds, three carotenoids, and twenty-nine lipid molecular species were identified. The (poly)phenol extract from purple Polignano carrots induced a dose-dependent hormetic response in the range 50–500 μg/mL in HT-29 and U2OS cancer cells, stimulating viability at low concentrations and slightly reducing it at higher doses. In contrast, a carotenoid-enriched nanoemulsion triggered marked cytotoxic and pro-apoptotic effects in U2OS cells at 50 μg/mL concentration, as evidenced by increased caspase-3 activity, while a control pumpkin-derived nanoemulsion was inactive. These results highlight the distinct biological activities of (poly)phenols and carotenoids and underscore the importance of nanoformulation strategies in enhancing carotenoid bioactivity, supporting their potential application in functional foods and nutraceutical development.

## 1. Introduction

Fruits and vegetables contain various natural pigments, such as carotenoids, anthocyanins, and phenolic compounds, which have intriguing antioxidant and biological properties tested in multiple experimental models [1,2]. Among these, carrot (*Daucus carota* L.), a biennial vegetable of the Apiaceae family, is one of the most widely cultivated vegetables globally and is widely consumed due to their pleasant flavor and health benefits, which include high levels of phenols and (poly)phenols. Additionally, they are one of the best sources of carotenoids, with a content of up to 55 mg per 100 g, providing a significant amount of dietary provitamin A through their precursors (α and β-carotene) [3,4,5]. Furthermore, carrots contain many other beneficial compounds, including vitamins, minerals (notably high potassium levels), and dietary fiber. Additionally, black and purple varieties incorporate anthocyanins, conferring a higher nutritional value in comparison to commercially available orange varieties [6]. In recent decades, fruits and vegetables high in anthocyanins have gained popularity due to their health benefits. As interest in pigmented carrots grows, some purple and black varieties are becoming the focus of many research studies [7]. Black carrot is widely used as a natural source of anthocyanins for food coloring and functional extracts with antiradical radical scavenging and antioxidant properties [8,9,10].

The yellow-purple Polignano carrot is a multicolored landrace variety of carrot cultivated in the Apulia region of southern Italy. Its cortex pigmentation ranges from yellow or deep orange to dark purple, while the inner core varies from pale yellow to light green [11]. Due to their unique color, sweetness, and distinctive cultivation and harvesting methods, these carrots have been included on the Slow Food list of traditional products by the Slow Food Association. Additionally, considering that the production of this carrot type is minimal, with a cultivation area of about 20 ha [12] and an annual output of about 4–5 t/ha, the yellow-purple Polignano carrot was also included in the Rural Development Program “P.S.R. 2007-2013” of the Italian Puglia County for biodiversity protection, as a species at risk of genetic erosion. Therefore, enhancing the quality and functional attributes of these distinctive carrots is essential to increase consumer demand and support production. This local landrace has a qualitative profile that is characterized by high concentrations of antioxidants (4 times higher than the commercial carrots), phenols, and carotenoids, especially β-carotene. This profile, combined with its low glycemic index, may account for the high nutritional quality of this landrace, especially the purple type [11]. Pace et al. [13] reported a deeper qualitative and biochemical characterization of fresh-cut purple Polignano carrots during cold storage in air or passive modified atmosphere. The study highlighted a rich composition in polyphenol compounds (belonging to the classes of hydroxycinnamic acids and their derivatives, and anthocyanins) and carotenoids, with β-carotene as the most represented, followed by lutein and α-carotene [13].

In this context, nanoemulsion systems represent a key technological approach to address the limited bioavailability and stability of lipophilic bioactive compounds, such as carotenoids, which constitute a major constraint for their effective use in nutraceutical and functional food applications. By reducing droplet size and increasing surface area, nanoemulsions improve the dispersion of hydrophobic molecules in aqueous environments, protect them from chemical degradation, and enhance their cellular uptake, ultimately leading to improved biological activity.

Therefore, the aim of this study was to investigate whether nanoemulsification could enhance the biological effects of bioactive compounds extracted from purple Polignano carrots, a variety characterized by high nutraceutical value. Specifically, this study represents the first evaluation of the cytotoxic, apoptotic, and antiproliferative effects of (poly)phenols, carotenoids, and lipids extracted from purple Polignano carrots (pPC), comparing their activity in both non-nanoemulsified and nanoemulsified forms. To this end, human cell lines HT-29 (colon adenocarcinoma) and U2OS (osteosarcoma) were employed as experimental models. Although these preclinical models have not previously been used to assess the biological activity of pPC, they are well recognized for their resistance to cell death stimuli [14,15] and have been widely used to evaluate the cytotoxic, apoptotic, and autophagic effects of polyphenols [16] and carotenoid-rich matrices [17].

The central research hypothesis is that the exceptionally high antioxidant capacity and unique phytochemical profile of the purple Polignano carrot confer dose-dependent and formulation-dependent biological effects, including hormetic responses mediated by (poly)phenols and pro-apoptotic activity mediated by carotenoids delivered via nanoemulsions. Completion of these aims will contribute to establish the purple Polignano carrot as a high-value functional food source, providing mechanistic evidence supporting the rational design of dietary interventions and nanoformulated nutraceuticals. Ultimately, this work will advance the understanding of how natural phytochemical complexity, dose, and delivery systems interact to modulate cellular health.

## 2. Materials and Methods

### 2.1. Chemical and Reagents

Methanol, 2,2-diphenyl-1-picrylhydrazyl (DPPH), and 6-hydroxy-2,5,7,8-tetramethylchroman-2-carboxylic acid (Trolox) were purchased from Merck/Sigma-Aldrich (Milan, Italy). Methanol, acetonitrile, acetone, ethyl acetate (all HPLC grade), and formic acid were obtained from Merck Millipore (Darmstadt, Germany). 3-*O*-Caffeoylquinic acid (chlorogenic acid), cyanidin-3-*O*-glucoside chloride, lutein, and β-carotene were acquired from Extrasynthese (Genay, France). Caffeic acid, ferulic acid, and coumaric acid were obtained from Merck/Sigma-Aldrich. HPLC-grade water (18.2 MΩ) was prepared using a Millipore Milli-Q purification system (Merck Millipore, Darmstadt, Germany). All other chemicals, reagents, and solvents used in this study were of analytical reagent grade and purchased from Merck/Sigma-Aldrich (Milan, Italy).

### 2.2. Plant Materials

**Figure 1:**
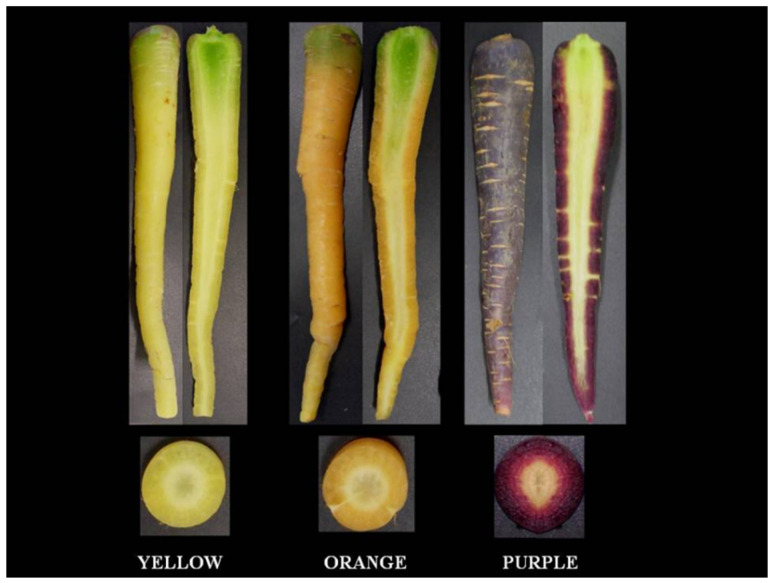
Representative image of the yellow, orange, and purple Polignano carrots.

Yellow-purple Polignano carrots (*Daucus carota* L. var. sativus) were purchased in 2024 from a local producer at the maturity stage proper for commercialization and transported in refrigerated conditions to the Postharvest Laboratory of the Institute of Food Production Sciences of CNR. Fresh samples were divided into three groups based on external color: yellow, orange, and purple, and processed separately. The carrots were brushed, washed in tap water, peeled, and dried; they were then sliced into uniform discs of approximately 3–5 mm thickness using a stainless-steel mandoline and used for subsequent analyses. An image of the yellow, orange, and pPCs is shown in Figure 1.

### 2.3. Antioxidant Activity of Yellow, Orange, and Purple Polignano Carrots

The antioxidant activity of the yellow, orange and purple typology was measured as previously described [18]. Briefly, 5 g of carrot slices were chopped into approximately 2–3 mm pieces using a stainless-steel knife and homogenized in 20 mL of a methanol/water solution (80:20 *v*/*v*) for 2 min using an Ultra-turrax homogenizer (T-25-Ika-Werke, Staufen, Germany). The mixture was then centrifuged (Prism C2500-R, Labnet, Edison, NJ, USA) at 6440× *g* for 5 min at 5 °C. Next, 50 μL of the extract was mixed with 950 μL of a 0.1 mM DPPH solution in ethanol, and the mixture was incubated for 30 min at room temperature in the dark. The absorbance was measured at 515 nm and compared to that of the non-inhibited DPPH using a spectrophotometer (UV-1800—Shimadzu, Kyoto, Japan). Data were expressed based on fresh weight and reported as milligrams of Trolox per 100 g, using a Trolox calibration curve (82–625 μM; R2 = 0.99).

### 2.4. Extraction and Analysis of Phenolic Compounds, Carotenoids, and Other Lipids from Purple Polignano Carrots

For phenol extraction, pPC slices (5 g) were finely chopped into approximately 2–3 mm pieces using a stainless-steel knife, then treated with 20 mL of 2% formic acid in a methanol/water (60/40 *v*/*v*) mixture for 1 h on a horizontal shaker in the dark for phenolic extraction, following a previously reported method [13]. The extraction process was repeated twice; supernatants were combined, dried using rotary evaporation (LaboRota 4000/HB Efficient, Heidolph, Schwabach, Germany), and stored at −20 °C until needed. Carotenoids and other lipids from 2.5 g of chopped samples were extracted using acetone until discoloration, as previously described [13]. After filtration, the extracts were dried under a stream of nitrogen and stored at −20 °C until further analysis. Phenolic compounds in the carrot extract were identified with HPLC coupled to multistage ion trap MS with electrospray ionization (HPLC/ESI-ITMS^n^), using a Surveyor MS micro HPLC connected to a Finnigan LCQ DECA XP Max mass spectrometer, managed by Xcalibur^®^ software 1.3 version (Thermo Finnigan, San Jose, CA, USA). The column effluent was split using an in-line T junction: 80% was directed toward a diode array detector (DAD) and 20% to the mass spectrometer. Chromatographic and mass spectrometric conditions followed those previously reported [13]. Briefly, phenols were separated on a XBridge BEH C18 column (130 Å, 5 mm, 2.1 mm × 150 mm, Waters) at a flow rate of 200 μL min^−1^ using water/acetonitrile/formic acid (96:4:0.1 *v*/*v*/*v*) as solvent A and water/acetonitrile/formic acid (45:55:0.1 *v*/*v*/*v*) as solvent B. After a 2 min hold at 6% solvent B, elution was performed by a linear gradient from 6 to 20% solvent B in 20 min, from 20 to 40% solvent B in 15 min, from 40 to 60% solvent B in 5 min and from 60 to 95% solvent B in 5 min, followed by 20 min of maintenance. The capillary voltage was set at −16 V, the spray voltage was −3.5 kV, and the tube lens offset was −10 V in negative ion mode, while, in positive ion mode, the capillary voltage was set at 36 V, the spray voltage was 3 kV, and the tube lens offset was 45 V. The capillary temperature was 275 °C. Mass spectra were recorded from mass-to-charge ratio (*m*/*z*) 80 to 1500 both in negative and positive ionization mode and data were acquired in MS, MS/MS, and MS^n^ scanning modes. To analyze carotenoids and other lipids, the same HPLC-IT-MS^n^ system equipped with an atmospheric pressure chemical ionization (APCI) source was used. For both analyses, chromatographic and mass spectrometric conditions followed those previously reported [13]. In particular, carotenoids separation was carried out using the C18 chromatographic column already described for phenolic compounds analysis by a 60 min’ isocratic elution with acetonitrile/methanol/ethyl acetate (80/17/3 *v*/*v*/*v*) at a flow rate of 200 μL min^−1^. Data were recorded in the 400–2000 *m*/*z* range in positive ionization mode and acquired in MS, MS/MS, and MS^n^ scanning modes. The APCI vaporizer temperature was set at 450 °C, the capillary voltage at 13 V, the discharge current at 5μA, and the tube lens offset at −15 V. The capillary temperature was 250 °C. Quantification of phenolic compounds and carotenoids was performed using HPLC-UV/Vis on an HP 1110 series HPLC (Agilent, Palo Alto, CA, USA) equipped with a binary pump (G-1312A) and an UV-Vis detector (G-1314A), following the analytical conditions previously described [13]. Briefly, individual phenols were separated on a XBridge BEH C18 column (130 Å, 5 mm, 4.6 mm × 150 mm, Waters) at a flow rate of 1 mL min^−1^; solvent A was water/acetonitrile/formic acid (95/4/1 *v*/*v*/*v*) and solvent B was water/acetonitrile/formic acid (44/55/1 *v*/*v*/*v*). The chromatographic conditions were as described for the HPLC-IT-MS^n^ system. Hydroxycinnamic acid derivatives were detected at 280 nm and anthocyanins at 520 nm. Quantification of phenolic compounds was performed with external calibration curves generated by repeated injections of appropriate standards prepared over different concentration ranges such as 1–300 μg mL^−1^ for each hydroxycinnamic acid standard and 0.1–200 μg mL^−1^ for cyanidin 3-*O*-glucoside to quantify all the anthocyanins, with six different concentration levels and duplicate injections at each level. In particular, caffeic acid hexoside, caffeoyl N-tryptophan and caffeoyl N-tryptophan hexoside were quantified using caffeic acid as reference; 3-O-caffeoylquinic acid and caffeoylquinic acid isomer were quantified using 3-O-caffeoylquinic acid (chlorogenic acid) as reference; ferulic acid hexoside, feruloylquinic acid and feruloyl N-tryptophan hexoside were quantified using ferulic acid as reference while 5-p-coumaroylquinic acid was quantified using coumaric acid as reference. All samples were prepared and analyzed in duplicate, and results were expressed as mg Kg^−1^ fresh weight (FW). For carotenoids quantification, acetone extracts were injected on the same C18 chromatographic column already described for hydroxycinnamic acids and anthocyanins analyses and separated by a 60 min’ isocratic elution with acetonitrile/methanol/ethyl acetate (80/17/3 *v*/*v*/*v*) at a flow rate of 1 mL min^−1^, monitoring the chromatogram at 490 nm. Quantification was carried out using external analytical curves of lutein and β-carotene (for both α-carotene and β-carotene quantification) over a concentration range of 1–50 μg mL^−1^ with six concentration levels and duplicate injections at each level. All samples were prepared and analyzed in duplicate, and results were expressed as mg Kg^−1^ FW.

### 2.5. Preparation of Carotenoid Nanoemulsions from Purple Polignano Carrots

PCN enriched with carotenoid extracts from purple Polignano carrots were prepared using a high-energy emulsification-evaporation method [17,19]. Briefly, the carotenoid-rich extract obtained as described above was mixed with tetrahydrofuran (THF) in a 1:3 (*v*/*v*) ratio, containing 0.0025% (*w*/*v*) butylated hydroxytoluene (BHT) as an antioxidant. The addition of BHT was necessary to prevent oxidative degradation of carotenoids during the preparation process, as these compounds are highly susceptible to oxidation when exposed to light, oxygen, and organic solvents. The organic phase was then added dropwise to an aqueous solution containing 0.3% Tween 80, maintaining an organic-to-aqueous phase ratio of 1:9 (*v*/*v*). The resulting pre-emulsion was homogenized using an Ultra-Turrax homogenizer (T8 Ika-Werke) for three cycles of 2 min at 10,000× *g* [20]. Subsequently, to further reduce particle size and improve emulsion stability, the samples were subjected to ultrasonication with a Sonicator ultrasonic processor XL (Misonix, Farmingdale, NY, USA). Sonication was performed at 40% amplitude for three cycles of 30 s each, followed by one additional cycle of 40 s at 50% amplitude.

The residual THF solvent was removed by evaporation under a nitrogen stream for 30 min. The same solvent removal procedure was applied to control nanoemulsions (Ctrl/Veh), which were prepared by replacing the carotenoid extract with an equivalent volume of the THF/BHT solution, thereby ensuring identical processing conditions for both formulations. Sterilization of the final nanoemulsions was conducted under a sterile hood in laminar airflow conditions through filtration with a 0.2 μm membrane using sterile syringe filtration.

The carotenoid content incorporated into the nanoemulsions was quantified by extracting 0.5 mL of the emulsion with a hexane: ethanol (2:1 *v*/*v*) mixture, as described [17]. Quantification was performed spectrophotometrically by comparing the absorption spectra of the samples with those of carotenoids, using β-carotene as the reference standard and applying an external calibration curve.

Nanoemulsions were stored in the dark at 4 °C. Stability studies, including the maintenance of bioactive compounds and the assessment of biological activity, were conducted at 15-day intervals for a maximum period of one month. All nanoemulsion formulations were prepared in triplicate as independent experimental replicates.

### 2.6. Cell Lines and Biological Assays

The U2OS cell line, derived from human osteosarcoma [21], was obtained from the ATCC cell bank and kindly provided by Professor A. Oliva of the University of L. Vanvitelli in Naples, Italy. HT-29 cells [22], obtained from human colon adenocarcinoma, were acquired from an Italian distributor of ATCC cell bank, LGC Standards srl (Sesto San Giovanni, Italy). These cell lines have been routinely monitored for appropriate proliferation rates, doubling times, and mycoplasma contamination and cultured in Dulbecco’s Modified Eagle’s Medium (DMEM), supplemented with 10% fetal bovine serum (Euroclone, Pero, Italy), 1% L-glutamine, 1% penicillin, and 1% streptomycin (Euroclone). They were maintained at 37 °C in a humidified atmosphere with 5% CO2. The cells were harvested once they reached approximately 90% confluence. For viability tests, cells were plated in quadruplicate at a density of 2 × 10^4^ cells/mL with a total volume of 0.1 mL per well in a 96- well plate, allowing them to adhere for 24 h. Afterward, cells were incubated for an additional 72 h with various concentrations of phenolic compounds extracted from purple Polignano carrots (CPE). For PCN, incubation times ranged from 24 to 96 h, as specified. Cell viability was assessed using the Crystal Violet assay [23]. Briefly, the medium was carefully aspirated, and the cells rinsed with phosphate-buffered saline (PBS).

Following a 15 min fixation with 10% formalin at room temperature, 0.1% crystal violet (*w*/*v*) was added, and the cells incubated for 30 min at room temperature. The cells were then photographed under bright field (magnification 400×) with an inverted microscope (Axiovert 200 Zeiss, Jena, Germany). After washing, dye was solubilized with 10% acetic acid, and its absorbance measured spectrophotometrically at 590 nm. For both cellular models, the concentration of (poly)phenolic or carotenoid extracts required to reduce cell viability by 50% (EC_50_) was determined through dose–response analysis. Cells were subjected to treatment with the two types of extracts (as indicated in the figures) across a concentration range of 10 to 500 µg/mL (*w*/*v*), and EC_50_ values were derived by interpolating the dose–effect curves for cell viability (Supplementary data Figure S1).

To achieve an optimized, non-cytotoxic control of the vehicle used for resuspending the two extracts, control samples (Ctrl) were treated with the same non-cytotoxic volume of DMSO (<1% *v*/*v*) for CPE and with “empty nanoemulsions” for PCN at their maximum non-cytotoxic concentration (<5%).

To evaluate caspase-3 enzymatic activity, a proteolytic enzyme central to apoptosis signaling that degrades anti-apoptotic proteins such as the DNA repair enzyme Poly-ADP-Ribose-Polymerase (PARP), the cells were treated for 16 h with the specified PCN concentration. As a positive control, we used the combination of rTRAIL and quercetin, selected for its well-known apoptotic effect mediated by caspase-3 activation [24]. After treatment, cells were collected, centrifuged at 400× *g* for 5 min, washed with PBS, and lysed in a buffer (10 mM Hepes, pH 7.4; 2 mM EDTA; 0.1% [1]-1-propanesulfonate; 5 mM dithiothreitol; 1 mM phenylmethylsulfonylfluoride; 10 μg/mL pepstatin-A; 10 μg/mL aprotinin; 20 μg/mL leupeptin). The reaction buffer, combined with the conjugated amino-4-trifluoromethyl coumarin (AFC) substrate (Z-DEVD-AFC) from Euroclone, was mixed with 10 μg of protein from cell extracts and incubated at 37 °C for 30 min. Active caspase-3 cleaved the substrate (DEVD), releasing free AFC, whose fluorescence was measured using a multi-plate reader (Synergy HT BioTek, Milan, Italy) at an excitation of 400 ± 20 nm and an emission of 530 ± 20 nm. A standard AFC curve was used to quantify enzyme activity. Caspase-3 activity was expressed as nmol of AFC produced per min per μg of protein at 37 °C under a saturating substrate concentration of 50 μM [15].

### 2.7. Statistical Analysis

To compare the antioxidant activity of yellow, orange, and pPCs, a one-way ANOVA was performed on data means arranged in a completely randomized design, using the Student–Newman–Keuls (SNK) test. Additionally, the mean value ± standard deviation was calculated for all data presented.

Results of cellular assays are expressed as the mean ± standard deviation (SD) from two (2) or three (3) independent biological replicates, as specified. Each experiment was conducted in three technical replicates for caspase-3 activity or in four technical replicates for cell viability assays. The limitation of this analysis is that conclusions from experiments with n = 2 biological replicates should be interpreted conservatively. Where applicable, we emphasized consistency across independent experiments rather than relying on formal statistical analysis of significance. Specific values were indicated in figure legends: * *p* < 0.05, ** *p* < 0.01, *** *p* < 0.001; **** *p* < 0.0001” as calculated using the ordinary one-way ANOVA statistical test and multiple comparison corrected with Turkey’s post hoc analysis, by using Graph Prism 9 for MacOS.

## 3. Results and Discussion

### 3.1. Antioxidant Activity of Yellow, Orange, and Purple Polignano Carrots

Firstly, the antioxidant activity of pPCs was measured, yielding a value of 45 ± 0.23 mg Trolox g^−1^ FW, which was approximately ten times higher than that observed in the yellow and orange types (4.4 ± 0.3 and 5.0 ± 0.3 mg Trolox g^−1^ FW, respectively). These findings are consistent with previous reports, in which the antioxidant activities of Polignano carrots were tested [11,25].

Given the high antioxidant activity of pPCs, a more in-depth characterization of their (poly)phenolic, carotenoid, and lipid composition was performed. Subsequently, the biological effects of (poly)phenolic extracts from pPCs were evaluated on human colon adenocarcinoma (HT-29) and human osteosarcoma (U2OS) cell lines.

### 3.2. Phenolic Compounds, Carotenoids, and Lipid Composition in Purple Polignano Carrots

Based on the results from the antioxidant activity analysis, analyses of phenolic compounds, carotenoids, and lipids were subsequently conducted only on pPC extracts using HPLC-UV/Vis, HPLC-ESI-ITMS^n^, and HPLC/APCI-ITMS^n^. Regarding phenolic compounds, fifteen were characterized in the extract, and the classes identified matched those reported in our previous study [13] (Table 1). Specifically, ESI-ITMS^n^ identification confirmed the presence of hydroxycinnamic acids, their derivatives, and anthocyanins. Chlorogenic acid (3-*O*-caffeoylquinic acid) was the predominant hydroxycinnamic acids, while cyanidin 3-xylosyl (feruloylglucosyl) galactoside was the most abundant anthocyanin, accounting for 59.6% and 3.6% of the total phenolic compounds, respectively (Table 1).

Notably, cyanidin 3-xylosyl (feruloylglucosyl) galactoside makes up 70.2% of the total anthocyanins, aligning with previous findings where the phytochemical profile of different anthocyanin-rich carrots, a black carrot and a pPC, was compared to a commercial orange carrot [26], and the impact of cooking on pigmented bioactive constituents, particularly anthocyanins, in purple carrots was evaluated [27,28]. It is worth noting the presence of cinnamoyl- amino acid conjugates, first reported in the yellow-pPCs in our previous study [13], which account for 11.43% of the total phenolic compounds. More specifically, caffeoyl N-tryptophan hexoside, caffeoyl N-tryptophan, and feruloyl N-tryptophan hexoside account for 6.15%, 4.60%, and 0.68%, respectively (Table 1). The results of the quantitative analysis of phenolic compounds obtained in this study are in line with those of our previous studies on yellow-pPCs [13]. Hydroxycinnamic acids are mainly recognized as potent antioxidants and are involved in the prevention of several diseases connected to oxidative stress, such as cardiovascular and neurodegenerative diseases and cancer; furthermore, some hydroxycinnamic acids and their derivatives have also been recognized as having anti-inflammatory and antimicrobial properties [29].

Furthermore, the analysis and quantification of carotenoids in pPCs revealed the presence of lutein, α-carotene, and β-carotene, representing approximately 23.0%, 14.5%, and 62.5% of the total carotenoid content, respectively (Table 1). Carotenoids are a group of compounds that serve essential evolutionary functions in the life cycles of various organisms, such as the bright colors of birds, fish, insects, and more. Humans primarily obtain carotenoids through their diet, mostly from plant-based foods, but also from animal sources, such as eggs, salmon, and seafood. Given the various roles carotenoids play in plant tissue, as photo-absorbent pigments in photosynthesis and as protectors against foreign agents, due to their pro- and antioxidant properties, they have been linked to several biological effects in humans, as reported in numerous review articles [3]. Recent studies have shown that diets rich in carotenoids support a healthy gut microbiome [30]. This factor has often been ignored, but could be significant. This is because carotenoids largely bypass absorption in the small intestine and are transported to the colon, where they are partially degraded into metabolites that remain largely unidentified. These metabolites may include apo-carotenoids, which could exert biological effects due to increased aqueous solubility and electrophilicity, potentially enabling them to more effectively target transcription factors such as NF-κB, PPARγ, and RAR/RXRs. If absorbed in the colon, these compounds could exert both local and systemic effects. Carotenoids may also support the health of mucosal and gut barriers, including by stabilizing tight junctions [31,32].

**Figure 2:**
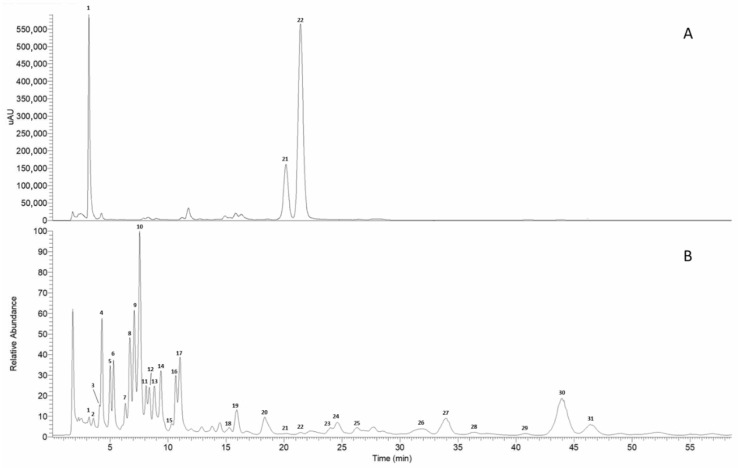
HPLC-Vis chromatogram recorded at 490 nm (**A**) and total ion current (TIC) chromatogram (**B**) from HPLC/APCI-ITMS^n^ analysis in positive ion mode of purple Polignano carrots acetone extract. The list of the identified compounds, including corresponding retention times, quasi-molecular ions and fragment ions, is shown in Table 2.

Liquid chromatography coupled with mass spectrometry using an APCI source (HPLC/APCI-ITMS^n^) also enabled a qualitative analysis of the complex lipid mixture present in the acetone extract. HPLC-Vis (490 nm) and total ion current (TIC) chromatograms obtained from the HPLC/APCI-ITMS^n^ analysis in positive ion mode of the acetone extract are shown in Figure 2 (Panels A and B, respectively). Using this method, a total of twenty-nine lipid molecular species, including neutral lipids (di- and triacylglycerols) and polar lipids (glycolipids, phospholipids, and sphingolipids), were identified (Table 2).

The lipid classes found in pPCs aligned with previous research [33]. Specifically, the glycolipids identified in the acetone extract of pPCs included four monogalactosyldiacyl-glycerols (MGDG) (34:6, 36:6, 36:5, and 36:4) and five digalactosyldiacylglycerols (DGDG) (34:6, 36:6, 36:5, 36:4, and 34:2), identified in the HPLC/APCI-ITMS^n^ analysis as [M + NH4]^+^ ions (Table 2). MGDG and DGDG are glycolipids with two fatty acyl groups attached at the sn-1 and sn-2 positions of the glycerol backbone, with one or two galactose units linked to the sn-3 position via acetal linkage. Several studies have shown that glycoglycerolipids possess specific biological activities, including antiviral, antitumor, and anti-inflammatory effects [34]. Moreover, due to the continuous rise in obesity and related diseases, in recent years there has been an increasing interest in effective appetite regulation methods and some studies indicate that the intake of DGDG could effectively inhibit the activity of lipase, an enzyme that breaks down triglycerides into free fatty acids and glycerol by catalyzing the hydrolysis of the ester bonds in triglycerides, slowing down the rate of fat breakdown and thereby contributing to appetite suppression [35]. Furthermore, four ceramides (t18:1-C16:0h, t18:1-C22:0h, t18:1-C24:0h, and t18:1-C25:0h), a class of lipids composed of a sphingoid base and a fatty acid, along with three cerebrosides (t18:1-C16:0h-Glu, d18:2- C16:0h-Glu, and t18:1-C24:0h-Glu), which consist of a ceramide attached to a sugar unit, were also identified. Sphingoid bases are indicated as t18:1 for 4-hydroxy-8-sphingenine and d18:2 for 4,8-sphingadienine. The phospholipid classes identified included phosphatidylcholines (PCs) such as (36:4) C18:2/C18:2, (36:3) C18:1/C18:2, and (34:2) C16:0/C18:2, a lipid class that features choline as its headgroup, and N-acylphosphatidylethanolamines (NAPEs) (36:6-N22:3, 36:6-N22:2, 36:6-N20:0, and 36:4-N22:2), triacylated phospholipids derived from phosphatidylethanolamine with an additional N-linked fatty acyl chain [36], detected as the molecular ion [M + H]^+^. Dietary phosphatidylcholines are the main source of choline, an essential nutrient that supports lipid and amino acid metabolism and contributes to cell membrane structure [37]. Furthermore, choline deficiency causes disorders, including hepatic abnormalities, and is associated with an increased risk of multiple types of cancer. A recent study demonstrated that the tumor suppressor TP53 drives the Kennedy pathway, which is associated with phosphatidylcholine metabolism, and that TP53 loss leads to dysregulated choline metabolism and perturbed cellular homeostasis, contributing to tumorigenesis [38].

### 3.3. Effects of (Poly)Phenols and Carotenoids from Purple Polignano Carrots on Cell Viability and Cell Death

Despite numerous recent studies on the health effects of carrot phytochemicals [28] no study has examined the chemical and biological characterization of two distinct components ((poly)phenols and carotenoids) of purple Polignano carrots in preclinical models of colon and osteosarcoma tumors. In this study, we compared for the first time two types of natural classes of molecules extracted from the same food matrix, belonging to CPE and PCN, to verify whether they possess the same functionality using biological assays. To investigate the biological effects of CPE and PCN from pPCs, two human cell lines were used: HT-29, derived from a human colon adenocarcinoma, and U2OS, derived from a human osteosarcoma. These cell lines were selected based on their high resistance to drug- or γ-radiation-induced cell death [15] and their known responsiveness to extracts enriched in (poly)phenols [16] and carotenoids [17,39] obtained from various food sources. In addition, these cell lines have been partially characterized genetically for the presence of either a wild-type form (U2OS) or a mutated, overexpressed form (HT-29) of the tumor suppressor protein TP53. Given this protein’s pleiotropic roles in cells, ranging from apoptosis to the cell cycle to metabolism, these differences could underlie the observed differential biological effects [40].

**Figure 3:**
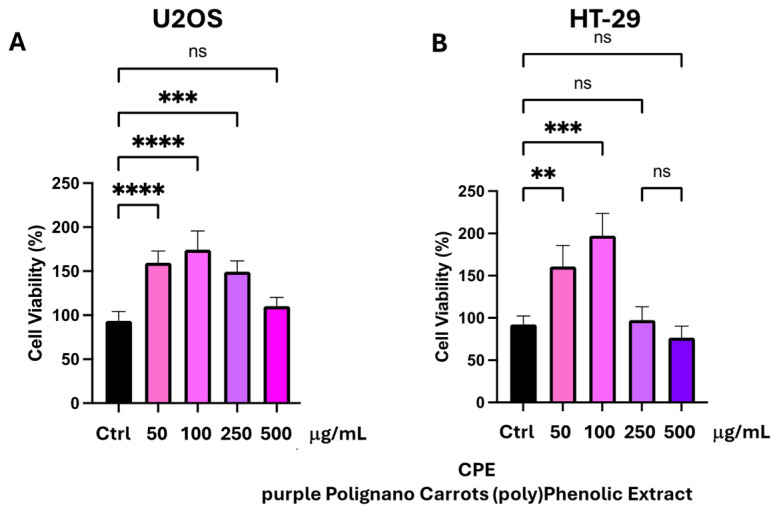
Cell viability of U2OS (**A**) and HT-29 cells (**B**) treated with increasing concentrations of purple Polignano carrot (poly)phenol extract (CPE) for 72 h. Viability was assessed by the Crystal Violet assay (absorbance at 595 nm) and expressed relative to untreated controls (Ctrl). The data represent two independent experiments performed in quadruplicate. Statistical significance vs. Ctrl: ** *p* ≤ 0.01, *** *p* ≤ 0.001, **** *p* ≤ 0.0001; ns = not significant (ANOVA).

As shown in Figure 3, CPE exerted only a modest effect on cell viability in both U2OS and HT-29 cells after 72 h of incubation. At lower concentrations (50–250 μg/mL, *w*/*v*), CPE significantly enhanced cell proliferation in U2OS cells compared with untreated controls (Ctrl; Figure 3A). The calculated half-maximal effective concentration (EC_50_) was approximately 7.5 mg/mL. A comparable proliferative effect was observed in HT-29 cells treated with CPE at concentrations of 50 and 100 μg/mL (*w*/*v*), with no significant changes in viability at higher concentrations (250–500 μg/mL). For HT-29 cells, the EC_50_ was approximately 0.75 mg/mL (Supplementary Figure S1), which is about 10-fold lower than that observed in U2OS cells (Figure 3B).

This differential effect of CPE on cell death resistance observed in these preclinical cancer cell models may also be attributed to the presence of wild-type TP53 in U2OS cells, as opposed to the mutated variant present in the HT-29 cell line. To substantiate this hypothesis, considering the distinctive role of TP53 as the “guardian of the genome,” we further assessed the heightened resistance to cell death induced by γ-rays in U2OS cells relative to SAOS cells, which lack TP53, and HT-29 cells [14,15].

The U-shaped cell viability pattern observed suggests hormesis, an adaptive stress response common to other phenolic compounds [41] in which low doses of a potentially harmful agent stimulate protective mechanisms, thereby increasing cellular resistance and survival. In the case of polyphenols, low concentrations often act as mild pro-oxidant or electrophilic signals, triggering redox-sensitive pathways rather than functioning as direct antioxidants. This controlled stress activates conserved cytoprotective mechanisms, including the Nrf2/ARE signaling axis, MAPK cascades, and mitochondrial stress responses, leading to enhanced antioxidant capacity, improved proteostasis, and increased cellular resilience. Such adaptive activation results in increased cell survival and metabolic competence within the hormetic zone [42]. At higher concentrations, however, the same polyphenols may overwhelm cellular defense systems by excessive ROS generation, disruption of mitochondrial function, or interference with protein and membrane integrity, thereby producing cytotoxic effects and reduced cell viability. This dual behavior explains the descending limb of the U-shaped response and highlights the dose-dependent balance between eustress and distress that underlies polyphenol bioactivity [43]. Overall, the observed U-shaped viability profile supports the view that polyphenols exert their beneficial effects primarily through hormetic signaling rather than direct antioxidant action, reinforcing the concept of polyphenols as adaptive response modifiers that enhance cellular stress tolerance when present at low, physiologically relevant concentrations [44].

Given these results and the absence of other biological parameters for evaluation in our next study, our explanation remains speculative but is well supported by the literature. For example, in a recent article [45], we used a very low dose of curcumin in myeloid cells. We observed a redox imbalance in the cells, followed by induction of autophagy and activation of the NRF2/ARE system, with increased expression of antioxidant genes, including NQO1 and HO-1. This study shows that, at low doses compatible with plasma concentrations, a natural polyphenol can stimulate a protective homeostatic response following a non-toxic redox imbalance, involving autophagy and antioxidant gene expression, thereby protecting cells against subsequent external stressors and environmental pollutants. Regarding cell viability, tested in this manuscript using an extract enriched with different classes of (poly)phenols, this hormetic response could be related to the synergistic effects of low doses of specific polyphenols and may be linked to proliferative behaviors.

In the context of cancer cells, this response has critical implications: while low concentrations of pharmacological agents or stressors might initially seem harmless or even beneficial (“eustress”), they can paradoxically enhance the resilience of malignant cells, potentially promoting survival, adaptation, and resistance (for example, autophagy) to subsequent treatments. In the context of natural compounds, such as (poly)phenolic extracts, this biphasic dose–response implies that while high doses of (poly)phenols exert cytotoxic effects, low doses may paradoxically promote cell viability by triggering protective cellular mechanisms such as antioxidant defenses, stress response pathways, or DNA repair. These findings underscore the importance of careful dose optimization in the development of (poly)phenol-based adjuvant anticancer therapies, as subtherapeutic levels might inadvertently enhance cancer cell resilience, potentially diminishing therapeutic efficacy or even contributing to treatment resistance [45].

To investigate the biological effects of the carotenoid fraction, an effective cellular delivery method was developed using oil-in-water (O/W) nanoemulsions prepared by a high-energy emulsification-evaporation technique, which enhances cellular uptake [17,46]. PCN derived from pPCs was employed. The nanoemulsion system was specifically developed as a carrier in the cell culture medium to enable the delivery of carotenoids, which are highly apolar, and to stabilize these chemically labile compounds. The THF employed was used as a solvent to develop these formulations due to its ability to dissolve the hydrophobic carotenoids and facilitate the formation of stable nanoemulsions. THF was completely removed by evaporation under a nitrogen stream prior to biological testing, as detailed in Section 2. Consequently, the final nanoemulsions administered to cells were solvent-free, rendering them suitable for further in vitro experimentation. For the development and future application of these formulations in the food and nutraceutical sectors, appropriate food-grade solvents would need to be employed. Notably, nanoemulsions containing carotenoids derived from pumpkin, a compositionally distinct vegetal matrix, had previously been evaluated on human colon adenocarcinoma and osteosarcoma cell lines [17]. This delivery system, already tested in our previous studies, proved to be significantly more effective in transporting hydrophobic compounds such as carotenoids compared to other methods, including DMSO dissolution or micelle preparations, which had also been explored. For this reason, and for the purposes of the present study, the carrier system demonstrated its ability to functionalize and deliver these compounds efficiently, representing a first step toward the development of food-grade formulations as outlined above. Based on these prior findings, the present experiments were conducted exclusively on U2OS cells to determine whether the biological effects observed with a different carotenoid source could be reproduced in an alternative cellular model. This strategic choice aimed to broaden the understanding of the potential bioactivity and cellular responsiveness to carotenoid- enriched nanoemulsions across diverse cell types.

**Figure 4:**
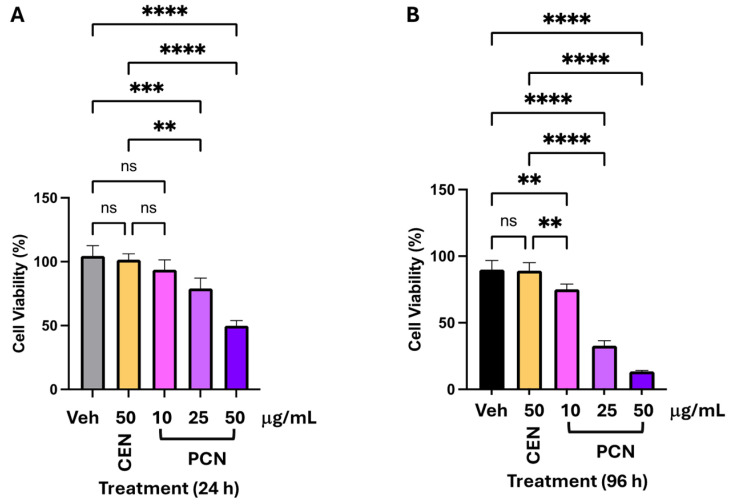

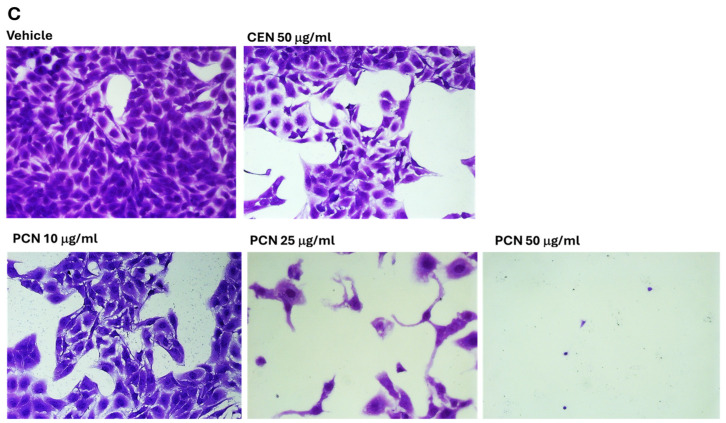
Viability of U2OS cells treated with carotenoid-enriched nanoemulsion from purple Polignano carrot. Cells were exposed to the indicated concentrations of PCN and CEN for 24 h (**A**) or 96 h (**B**). Viability was measured by the Crystal Violet assay and expressed relative to vehicle-treated controls (Veh). The data represent two independent experiments performed in quadruplicate. Statistical significance vs. Veh: ** *p* ≤ 0.01, *** *p* ≤ 0.001, **** *p* ≤ 0.0001; ns = not significant (ANOVA). (**C**) Representative micrographs of Crystal Violet–stained cells (200×, inverted microscope, Axiovert 200, Zeiss).

PCN applied at final carotenoid concentrations of 10–50 µg/mL (*w*/*v*) was significantly cytotoxic to U2OS cells, starting from 25 µg/mL. The calculated EC_50_ was about 50 µg/mL after 24 h of treatment, based on a cell viability dose-effects curve (Figure 4A; Supplementary Figure S1). Given the doubling time of these cells (approximately 30 h), we selected two time points (24 h and 96 h) to better evaluate the biological effect of PCN on cell viability. In fact, extending the incubation with PCN to 96 h, cytotoxicity, as expected, significantly increased in parallel with the EC_50_ value that was reduced to about 23.5 µg/mL (Figure 4B, Supplementary Figure S1). To further assess the cytotoxic effect of PCN, the cell viability experiments were performed using a carotenoid bioactive extract (CEN) derived from seeds and pulp of Cucurbita moschata (pumpkin) [17,39] as a positive control. This carotenoid-enriched extract, obtained by supercritical CO_2_ extraction and previously analytically characterized [47], was selected for its previously documented effects on two colon and osteosarcoma cell line models [17].

Compared with the PCN extract, β-Carotene appears to be the most representative carotenoid in CEN; however, when applying the same β-Carotene concentration to our cellular models (osteosarcoma cells), we did not observe the same phenotype (cell death or a delay in cellular proliferation). This observation further supports the hypothesis that the cellular effects may result from the synergistic presence of multiple bioactive molecules in the extracts, which cannot be recapitulated by a single molecule [17,39].

As reported in Figure 4, CEN did not exhibit any significant cytotoxic effects compared to PCN, even at extended incubation times. To confirm these data, Figure 4C provides a representative image of U2OS cells treated with the indicated concentrations of PCN and CEN after Crystal Violet staining. Considering the cytotoxic effect of PCN, we aimed to investigate whether it was linked to the activation of the programmed form of cell death, such as the apoptotic process. Therefore, apoptosis was assessed after 16 h of incubation of U2OS cells with 50 µg/mL PCN.

This timing course was selected prior to documenting extensive cell death in U2Os cells to prevent caspase-3 enzymatic activity from being a secondary effect of a necrotic phenotype [16]. Indeed, early time points (6 h) did not show an increase in caspase-3 activity in U2OS cells, as verified using a positive control involving a recombinant agonist of a cell death receptor (rTRAIL) and the natural flavonoid quercetin (Q), as demonstrated in previous studies involving preclinical models [24,48].

**Figure 5:**
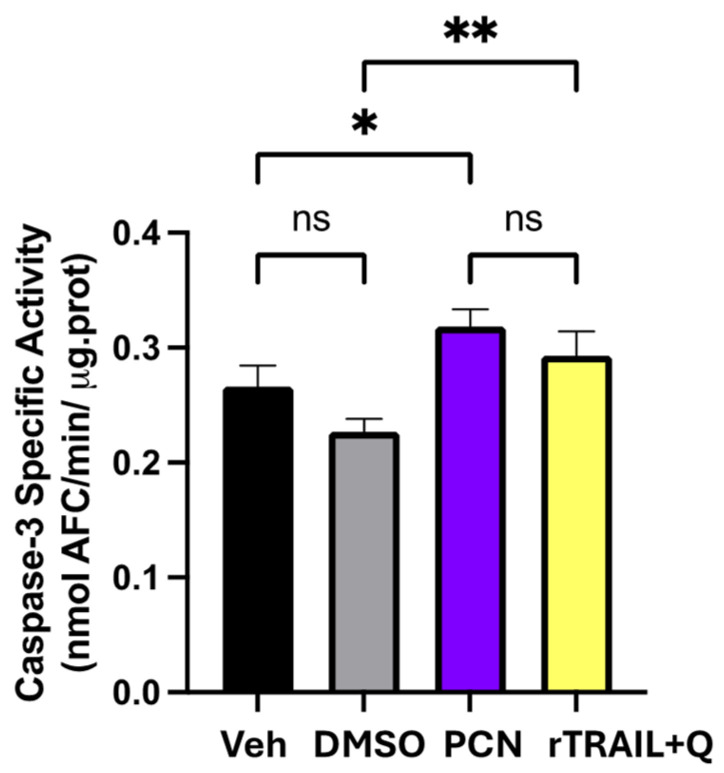
Caspase-3 activity in U2OS cells treated with carotenoid-enriched nanoemulsion from purple Polignano carrot. Cells were incubated for 16 h with PCN (50 µg/mL). As a positive control, cells were treated with rTrail + Q (10 ng/mL and 50 μM, respectively). Caspase-3 activation was quantified using the DEVD-AFC fluorometric substrate and expressed as specific enzymatic activity. The data represent two independent experiments performed in triplicate. Statistical significance vs. respective controls (Veh for PCN and DMSO for rTrail + Q): * *p* ≤ 0.05, ** *p* ≤ 0.01, ns = not significant (ANOVA).

Elevated caspase-3 enzymatic activity is a well-established marker of apoptosis and is the primary assay used in our studies to demonstrate apoptosis induction [24,48]. In fact, caspase-3, a cysteine protease that cleaves and degrades a variety of anti-apoptotic proteins, is a pivotal element of apoptotic signaling. This enzyme facilitates apoptosis through both the extrinsic pathway (involving death receptors such as FasR/TrailR1-2) and the intrinsic (mitochondrial-dependent) pathway. For these reasons, a positive control was established using the combined treatment with the death ligand (rTrail) and the natural flavonoid quercetin (rTrail + Q) [24,48]. As demonstrated in Figure 5, treatment with PCN resulted in a statistically significant increase (20%) in caspase-3 activity compared with untreated cells. This level of caspase-3 activation, even if not impressive, was comparable to that observed with the rTrail + Q treatment, indicating that a combination of bioactive carotenoids (lutein, α-, β-carotene) and lipid compounds, such as sphingolipids [49], present in pPCs, may exert additive or synergistic effects in inducing apoptotic cell death in the U2OS cell line.

The elevated cell death measured in our cell viability assay after 24 h compared to the level of caspase-3 activity does not exclude the possibility that, as always verified in a cellular context, it could be attributable to different forms of cell death, such as lethal autophagy or necroptosis, coexisting in the same context and dependent on the heterogeneity of the cellular status (such as cell cycle phase or cellular type or death stimuli) [15,39].

In future studies, we may also assess other apoptotic markers, such as PARP cleavage and DNA fragmentation, or whether a lethal variant of autophagy (programmed cell death type 2) might contribute to the cytotoxic effects of PCN in U2OS osteosarcoma cells, analogous to the findings with CEN in a comparable osteosarcoma cell line model (SAOS) [39]. However, these biochemical markers exhibit different kinetic profiles of appearance, and, to the best of our knowledge, caspase-3 activation is among the earliest apoptotic markers to evaluate in a cellular context.

To resume the biological assay findings, although the precise molecular mechanisms underlying the divergent effects of CPE and PCN were not fully elucidated in the present work, we propose plausible hypotheses that warrant further investigation. One such hypothesis posits that modulation of autophagy is a key contributor to the protective and context-dependent effects of natural bioactive compounds, including carotenoids and polyphenols, in transformed cell models. This notion is supported by previous studies demonstrating that these molecules can differentially regulate autophagy- and survival-related pathways depending on cell type and experimental conditions [16,17]. Importantly, these effects appear to be highly dependent on both the cellular context (presence or absence of a specific tumor suppressor protein) and the specific phytochemical composition of the extract employed.

Accordingly, our ongoing and future studies will focus on dissecting the molecular targets and signaling pathways involved in the regulation of autophagy and apoptosis, particularly the PI3K/AKT and mTOR/AMPK axes, mediated by (poly)phenol- and carotenoid-rich purple carrot extracts in U2OS and HT-29 cell lines. A limitation of our study is that, at this stage, we cannot identify the specific molecular target or bioactive molecule responsible for the observed biological effects. Future studies should focus on isolating and characterizing the active compound(s) and defining the molecular mechanism(s) underlying these effects, which will be essential for confirming causality and assessing translational potential.

CPE primarily comprises molecules from the hydroxycinnamic acid family and the anthocyanin class (cyanidin derivatives; see Table 1). Concurrently, the carotenoid-derived matrix predominantly contains β-carotene and lutein (refer to Table 1).

Recent research has demonstrated that the health-promoting effects of anthocyanins, primarily cyanidin derivatives found in the purple carrot cultivar ‘Purple Sun’, may be linked to antioxidant activity in non-transformed cells (cultured myoblasts) and to proliferative arrest in various cancer cell lines, including colon cancer (HCT116) and leukemia (U937) [28]. However, cellular effects of this specific polyphenolic extract on cancer cells were observed only following administration of a considerably higher concentration, approximately ten times greater than that used in our assays (2.5 and 5.0 mg/mL versus the maximum dose of 0.5 mg/mL, see Figure 3). Notably, the authors employed docking simulation studies to identify potential molecular targets of cyanidin responsible for the antiproliferative effects observed.

Interestingly, the study reported lower binding energy between cyanidin and the sirtuin family member SIRT6, suggesting that cyanidin is a potent activator of SIRT6 and supporting its potential protective effects against metabolic and aging-related diseases [28]. The researchers demonstrated that cyanidin exhibits high specificity for the agonist-binding site of SIRT6; if this is confirmed in vivo, such specificity could also explain the observed proliferative behavior of CPE in the cancer cell lines utilized in this study (see Figure 3). This hypothesis is based on the evidence that low doses of resveratrol, a natural polyphenol with anti-aging properties, increase NAD+ levels, thereby activating the sirtuin family member SIRT1 [50].

Considering that dysregulated cancer cell metabolism is propelled by intracellular NAD+ concentrations supporting deacetylation mediated by sirtuins and ADP-ribosylation facilitated by PARP proteins, the possible impact of CPE on sirtuin activation (whether direct or indirect) may additionally affect the resistance of cancer cells to programmed cell death subsequent to DNA damage [50].

These hypotheses, based on the experimental observations detailed here (see Figure 3), deserve further study to identify the specific molecular target(s) of CPE. Additionally, they emphasize the need to carefully verify claims about the health benefits of the (poly)phenolic fraction of Polignano purple carrots. Nutraceuticals or supplements should be designed according to the specific dosage intended for use, including a polyphenolic extract from this food source at roughly ten times the amount used in our experiments, based on the EC_50_ value from cell viability tests (7.5 mg/mL in U2OS cells). It is also important to recognize that, from our experience, using a single representative molecule, such as β-carotene from pumpkin carotenoid extract or gallic acid from red wine polyphenolic extract, at a concentration equivalent to that in the food matrix does not replicate the biological effects seen with the whole extract in cellular models [16,17,39]. This is likely because the biological activity and molecular targets of the extract result from synergistic interactions among various natural compounds within the food matrix and across interconnected signaling pathways [16,17,39].

## 4. Conclusions

This study provides an in vitro chemical and biological evaluation of (poly)phenol- and carotenoid-rich extracts from Polignano carrots. Using cancer cell models, the two phytochemical classes showed markedly different biological behaviors: the (poly)phenolic extract (CPE) induced a hormetic-like, proliferative response in both U2OS and HT-29 cells, whereas the carotenoid nanoemulsion (PCN) exerted strong, dose- and time-dependent cytotoxic and pro-apoptotic effects, particularly in U2OS cells. The progressive reduction in EC_50_ values and the early activation of caspase-3 highlight apoptosis as a key mechanism underlying PCN-induced cytotoxicity. The nanoemulsion strategy was essential to enhance carotenoid solubility and cellular activity, as supported by comparison with a non-cytotoxic control nanoemulsion. Overall, the in vitro results demonstrate a clear differential response between (poly)phenolic and carotenoid fractions, underscoring the relevance of formulation strategies in modulating biological activity and supporting the anticancer potential of carotenoid-based nanoformulations.

This study presents several limitations primarily related to its in vitro design. First, the biological effects of the (poly)phenol- and carotenoid-rich extracts were evaluated exclusively in established cancer cell lines, which do not fully recapitulate the complexity of tumor microenvironments, systemic metabolism, or cell–cell interactions occurring in vivo. Consequently, the observed cytotoxic, proliferative, and pro-apoptotic responses may not directly translate to physiological or clinical conditions. Second, while nanoemulsification enhanced carotenoid bioactivity in vitro, the study did not address formulation stability under physiological conditions or potential effects of digestion and metabolism, which are critical determinants of bioavailability in vivo. Despite these limitations, the findings provide a robust in vitro foundation for future mechanistic studies and preclinical evaluations aimed at validating the anticancer potential of these formulations. Further mechanistic and preclinical studies are warranted to extend these findings.

## Figures and Tables

**Table 1:** Composition and content of phenolic compounds and carotenoids in Polignano purple carrots determined by HPLC-ESI-ITMS^n^ and HPLC-UV/Vis.

Phenolic Compounds	mg kg^−1^ FW
Caffeic acid hexoside	2.15 ± 0.41
3-*O*-Caffeoylquinic acid (Chlorogenic acid)	66.21 ± 1.92
Caffeoylquinic acid isomer	4.06 ± 0.97
Ferulic acid hexoside	3.73 ± 0.83
5-p-Coumaroylquinic acid	10.59 ± 0.78
Feruloylquinic acid	5.94 ± 0.35
Caffeoyl N-tryptophan	5.11 ± 0.29
Caffeoyl N-tryptophan hexoside	6.83 ± 1.06
Feruloyl N-tryptophan hexoside	0.76 ± 0.07
**Total hydroxycinnamic acids and derivatives**	**105.38 ± 2.80**
Cyanidin 3-xylosyl(glucosyl)galactoside	0.31 ± 0.06
Cyanidin 3-xylosylgalactoside	0.61 ± 0.07
Cyanidin 3-xylosyl(sinapoylglucosyl) galactoside	0.37 ± 0.06
Cyanidin 3-xylosyl(feruloylglucosyl) galactoside	3.99 ± 0.68
Cyanidin 3-xylosyl(coumaroylglucosyl) galactoside	0.37 ± 0.02
Pelargonidin 3-xylosyl(feruloylglucosyl) galactoside	0.05 ± 0.01
**Total anthocyanins**	**5.68 ± 0.89**
**Total Phenolic Contents**	**111.06 ± 2.13**
**Carotenoids**	
Lutein	1.63 ± 0.11
α-carotene	0.93 ± 0.09
β-carotene	3.87 ± 0.51
**Total Carotenoid Contents**	**6.43 ± 0.50**

**Table 2:** List of carotenoids and lipids identified in purple Polignano carrots acetone extract by HPLC/APCI-ITMS^n^. Specific quasi-molecular ions and fragment ions are reported for each compound.

Peak	t_R_ (Min)	*m*/*z*	MS/MS Ions *m*/*z*	Proposed Structure
**1**	3.19	569 [M + H]^+^	MS^2^ [569]:551, 533, 459	Lutein
**2**	3.58	926 [M + NH_4_]^+^	MS^2^ [926]:585; 567	DGDG (34:6)
**3**	4.11	764 [M + NH_4_]^+^	MS^2^:585; 567	MGDG (34:6)
**4**	4.29	954 [M + NH_4_]^+^	MS^2^ [954]:613, 595	DGDG (36:6)
**5**	5.00	792 [M + NH_4_]^+^	MS^2^ [792]:613, 595	MGDG (36:6)
**6**	5.30	956 [M + NH_4_]^+^	MS^2^ [956]:615, 597	DGDG (36:5)
**7**	6.32	794 [M + NH_4_]^+^	MS^2^ [794]:615, 597	MGDG (36:5)
**8**	6.73	958 [M + NH_4_]^+^	MS^2^ [958]:617, 599	DGDG (36:4)
**9**	7.10	732 [M + H]^+^	MS^2^ [732]:714, 570, 552	Cerebroside t18:1-C16:0h-Glu
**10**	7.56	782 [M + H]^+^	MS^2^ [782]:599, 319	PC (36:4) C18:2/C18:2
**11**	8.12	796 [M + NH_4_]^+^	MS^2^ [796]:617, 599	MGDG (36:4)
**12**	8.40	714 [M + H]^+^	MS^2^:696, 534, 516	Cerebroside d18:2-C16:0h-Glu
**13**	8.83	570 [M + H]^+^	MS^2^ [570]:552, 534	Ceramide t18:1-C16:0h
**14**	9.39	934 [M + NH_4_]^+^	MS^2^ [934]:593, 575	DGDG (34:2)
**15**	10.30	784 [M + H]^+^	MS^2^ [784]:601, 321, 319	PC (36:3) C18:1/C18:2
**16**	10.66	758 [M + H]^+^	MS^2^ [758]:575, 319, 295	PC (34:2) C16:0/C18:2
**17**	11.02	617 [M + H]^+^	MS^2^ [617]:599	DG (36:4)
**18**	15.26	619 [M + H]^+^	MS2 [619]:601	DG (36:3)
**19**	15.94	593 [M + H]^+^	MS2 [593]:575	DG (34:2)
**20**	18.33	1052 [M + H]^+^	MS^2^ [1052]:613	NAPE 36:6-N22:3
**21**	20.19	537 [M + H]^+^	MS^2^ [537]:481, 413, 401, 399	α-carotene
**22**	21.42	537 [M + H]^+^	MS^2^ [537]:481, 413, 401, 399	β-carotene
**23**	23.98	654 [M + H]^+^	MS^2^ [654]:618	Ceramide t18:1-C22:0h
**24**	24.60	1054 [M + H]^+^	MS^2^ [1054]:613	NAPE 36:6-N22:2
**25**	26.30	844 [M + H]^+^	MS^2^ [844]:682, 664, 646	Cerebroside t18:1-C24:0h-Glu
**26**	31.89	873 [M + H]^+^	MS^2^ [873]:855, 597, 595, 593	TG (54:9) C18:3/C18:4/C18:2 and C18:4/C18:1/C18:4 and C18:3/C18:3/C18:3
**27**	33.98	682 [M + H]^+^	MS^2^ [682]:664, 646	Ceramide t18:1-C24:0h
**28**	36.43	1030 [M + H]^+^	MS^2^ [1030]:613	NAPE 36:6-N20:0
**29**	40.80	883 [M + H]^+^ 696 [M + H]^+^	MS^2^ [883]:865, 601 MS^2^ [696]:678, 660	TG (54:6) C18:1/C18:2/C18:1 and C18:2/C18:2/C18:0 Ceramide t18:1-C25:0h
**30**	43.93	875 [M + H]^+^	MS^2^ [875]:857, 597, 595	TG (54:8) C18:3/C18:3/C18:2 and C18:3/C18:4/C18:1 and C18:4/C18:2/C18:2
**31**	46.40	1058 [M + H]^+^	MS^2^ [1058]:617	NAPE 36:4-N22:2

Abbreviations: MGDG, monogalactosyl diacylglycerol; DGDG, digalactosyl diacylglycerol; PC, phosphatidyl choline; DG, diacylglycerol; TG, triacylglycerol; d18:2, 4,8-sphingadienine; t18:1, 4-hydroxy-8-sphingenine; C16:0, palmitic acid; C18:0, stearic acid; C18:1, oleic acid; C18:2, linoleic acid; C18:3, linolenic acid; C18:4, stearidonic acid; C16:0h, 2- hydroxypalmitic acid; C22:0h, 2-hydroxybehenic acid; C24:0h, 2-hydroxylignoceric acid; C25:0h, 2-hydropentacosanoic acid; Glu, glucose; NAPE, N-acylphosphatidylethanolamine.

## Data Availability

The original contributions presented in the study are included in the article/Supplementary Materials, further inquiries can be directed to the corresponding author.

## Supplementary Materials

The following supporting information can be downloaded at: https://www.mdpi.com/article/10.3390/foods15091586/s1, Figure S1: EC_50_ calculation for PCN and PCE.

## Abbreviations

The following abbreviations are used in this manuscript:

pPC	purple Polignano carrot
CPE	(poly)phenol extract from purple Polignano carrot
PCN	carotenoid-enriched nanoemulsion from purple Polignano carrot
CEN	carotenoid-enriched nanoemulsion from pumpkin
DPPH	2,2-diphenyl-1-picrylhydrazyl
THF	tetrahydrofuran
BHT	butylated hydroxytoluene
