# Supplementary material for: Chemical Composition and Biological Activity of Phenolic Compounds and Carotenoids Extracted from Yellow-Purple Polignano Carrots
Source: Foods. 2026 May 4;15(9):1586. doi: 10.3390/foods15091586 (PMC13164324; doi:10.3390/foods15091586)
Supplement: Supplementary file 1 [file foods-15-01586-s001.zip › foods-4241352-supplementary.pdf]

Supplementary Figure S1-a-b-c-d

EC_{50} Calculation for PCN and CPE

a b

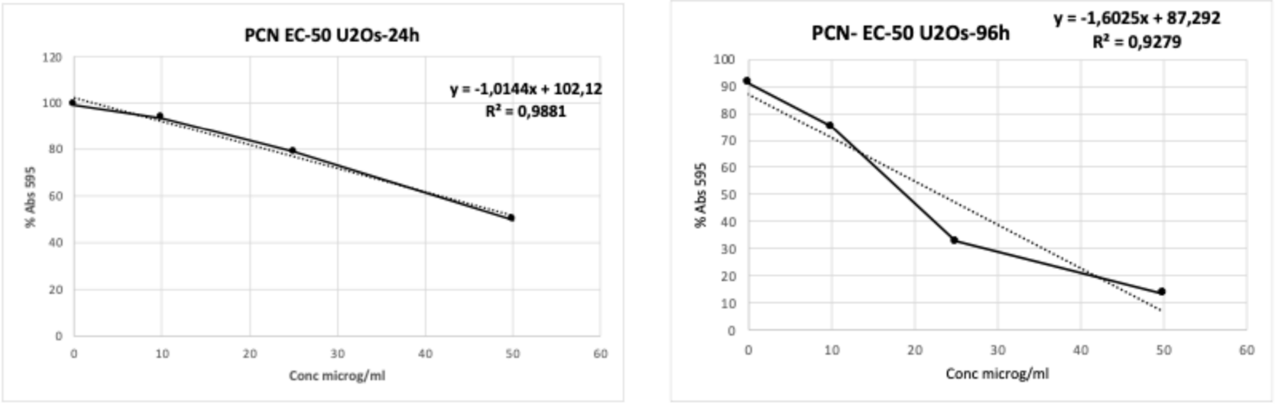

S1 The concentration of purple carotenoid extracts (PCN) required to reduce cell viability by 50% (EC_{50}) was determined through dose–response analysis. U2OS cells were subjected to treatment with the extracts (PCN as indicated in the figures) across a concentration range of 10 to 50 μg/mL (w/v), and EC_{50} values were derived by interpolating the dose–effect curves for cell viability (% Abs 595 nm) after 24 h (a) or 72 h (b).

c d

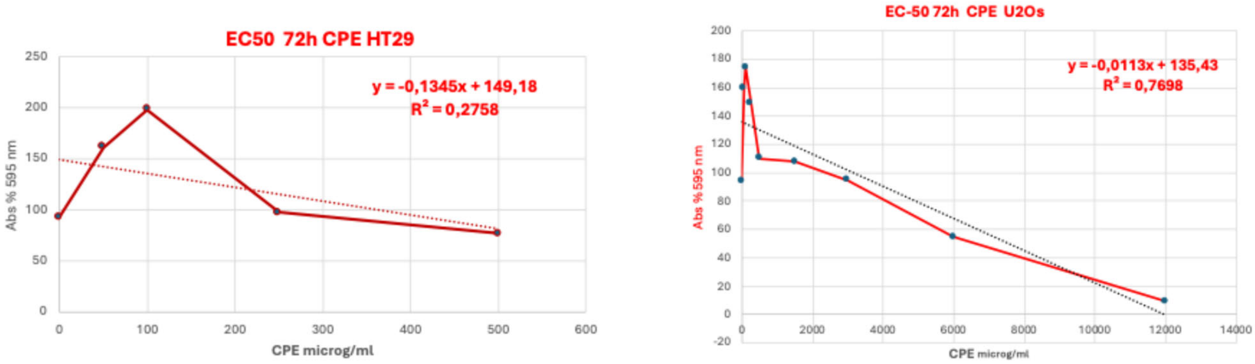

S1 The concentration of (poly)phenolic (CPE) required to reduce cell viability by 50% (EC_{50}) was determined through dose–response analysis. Cells were subjected to treatment with the extracts (CPE as indicated in the figures) across a concentration range of 50 to 500 for HT-29 (w/v), (c) or 50 to 1200 μg/mL for U2OS (d) and EC_{50} values were derived by interpolating the dose–effect curves for cell viability (% Abs 595 nm).
